# A Convenient RP-HPLC Method for Assay Bioactivities of Angiotensin I-Converting Enzyme Inhibitory Peptides

**DOI:** 10.5402/2013/453910

**Published:** 2012-10-21

**Authors:** Wei Wang, Nan Wang, Yu Zhang, Zheng Cai, Qihe Chen, Guoqing He

**Affiliations:** ^1^Institute of Quality Standards for Agriculture Products, Zhejiang Academy of Agricultural Science, Hangzhou 310021, China; ^2^College of Biology and Environmental Engineering, Zhejiang Shuren University, Hangzhou 310015, China; ^3^Departmentof Food Science and Nutrition, Zhejiang University, Hangzhou 310058, China

## Abstract

A convenient and accurate reversed-phase high-performance liquid chromatography (RP-HPLC) method for angiotensin I-converting enzyme inhibitory peptides assay was described in this paper. The mobile phase consisted of 70% A (0.05% TFA and 0.05% triethylamine in water, pH = 2.9–3.3) and 30% B (100% acetonitrile) using an Isogradient program. The flow rate was 0.5 mL/min. The absorb wavelength was 226.5 nm; the column temperature was controlled at 25°C. This method for angiotensin I-converting enzyme inhibitory peptides assay was convenient for the Iso-gradient program. The accuracy of the RT-HPLC method was verified by analyzing ACE inhibitory activity of the hydrolysate peptides of silkworm pupae protein, and the results showed that the RT-HPLC method was available for exploring new source of angiotensin I-converting enzyme inhibitory peptides rapidly and veraciously.

## 1. Introduction

Angiotensin-converting enzyme (ACE) is a di-peptidyl carboxypeptidase (EC 3.4.15.1) associated with the blood pressure regulation system of renin-angiotensin. This enzyme can increase blood pressure by converting decapeptide angiotensin I into potent vasoconstricting octapeptide angiotensin II, which leads to a consistent increasing of blood pressure. ACE has been recognized as critical in the renin-angiotensin-aldosterone system (RAAS) for leading to hypertension [1].

Over the last reports, the first ACE peptide inhibitor was discovered from snake venom due to its significant effects on the hypertension. Afterwards, more and more potent synthetic inhibitors of ACE, such as captopril and enalapril, were found continuously. Currently, the application of ACE peptide inhibitors has become an important way to cure hypertension, congestive heart failure (CHF), and chronic renal disease [2], but its side effects to the health are also noticeable [3]. Therefore, the bioactive peptides with ACE inhibitory activity were paid more and more attentiones because of their curative and nontoxic characteristics, especially the food-derived ACE inhibitory peptides, isolated from food or enzymatic digestion of food proteins, such as from gelatin [4], casein [5], fish [6], fig tree latex [7], *α*-zein [8], cereals and legumes [9], fermented soybean food products [10], soy protein [11], edible mushrooms [12], and microbes [13] that have been successfully used. These anti-ACE peptides derived from food protein hydrolysates might be used as main ingredients of blood pressure-lowering functional foods and nutriments [14].

In order to facilitate the identification and isolation of peptides with anti-ACE properties and explore extensive source of peptides with ACE inhibitory activity, a convenient and accurate method for ACE inhibitory activity assay *in vitro* was developed in this paper. 

At present, the method of Cushman and Cheung [15] was generally used to detect angiotensin I-converting enzyme inhibitory activity *in vitro *[1]. The principle of the method of Cushman and Cheung was ACE hydrolyzed substrate hippuryl-_L_-histidyl-_L_-leucine (HHL), then hippuric acid (HA) and histidyl-leucine (HL) was released, which could be extracted into ethyl acetate layer and detected at 228 nm [14]. However, the extraction was easily contaminated by HHL which was the weakness point of this method. Furthermore, other methods, such as fluorimetry [16–18], high-performance liquid chromatography (HPLC) [19, 20], and internally quenched fluorogenic methods [21], were quite complicated and exigent. Wu et al. [14] reported a direct HPLC analytical method and facilitated the assay of ACE inhibitory activity in some degree, but its elution conditions were gradient with several steps to adjust the elution conditions. This paper aims to set a RP-HPLC method with Iso-gradient elution conditions and explore the optimal quantity between HHL and ACE in the reaction system for getting a highest transform rate of HHL to HA. Furthermore, this novel RP-HPLC method is verified by analyzing the hydrolyzates of silkworm pupae protein.

## 2. Materials and Methods

### 2.1. Reagents

Angiotensin I-converting enzyme (0.25 U) and hippuryl-_L_-histidyl-_L_-leucine (HHL) were purchased from Sigma-Aldrich, USA. Hippuric acid (HA) and captopril were from Fluka, India. HPLC-grade trifluoroacetic acid (TFA), triethylamine (TTA), and acetonitrile were obtained from Tianjin Shield Company, China. Acidic protease (50000 U/g,* Aspergillus usamii NO. 537*) was from Wuxi enzymes of China. All other chemicals were of analytical grade.

### 2.2. Reagents Preparation for HPLC Analysis

HHL and ACE were dissolved in 100 mM borate buffer (pH = 8.3) supplemented with 300 mM NaCl, and their concentrations were 5 mM and 0.1 U/mL, respectively. Meantime, HA and Captopril (standard ACE inhibitor) were dissolved in distilled water.

The reaction system:10 *μ*L HHL, 10 *μ*L ACE, 40 *μ*L ACE inhibitors and 40 *μ*L 100 mM borate buffer (pH = 8.3). The system was incubated at 37°C for 30 min, and then 250*μ*L HCl was added. 

### 2.3. HPLC Analyze Conditions

The RP-HPLC system was consisted of a HPLC (2690 Waters, Milford, MA, USA) equipped with a Symmetry C_18_ column (4.6 × 150 mm, 5 *μ*m, Waters), 2996 photodiode array detector (DAD), system Instrument control, data collection, and Empower software of Waters Corporation. 

The mobile phase was solvent A, 0.05% TFA and 0.05% triethylamine in water (pH value at 2.9~3.3); solvent B, 100% acetonitrile; the ratio of solvent A/solvent B was 7/3. The flow rate was 0.5 mL/min, and the injection volume was 10 *μ*L. The elute was analyzed at a wavelength of 226.5 nm, which was at the maximum absorbance of HA, and column temperature was maintain at 25°C.

### 2.4. Protein Hydrolysate Preparation

The degreased dry silkworm pupae protein was hydrolyzed by acidic protease (50000 U/g, from* Aspergillus usamii No. 537*) at 35°C and pH 3.0 for 5 hours in a protein concentration 10%. Then the solution was filted by 0.45 *μ*m nylon syringe filter. Then, the supernatant was freeze-dried for HPLC analysis.

### 2.5. The Principle of the Convenient RP-HPLC Method

The substrate HHL showed a wider absorbance from 217 nm to 236 nm, and HA with the highest absorbance at 226.5 nm. At the optimum separation condition of the two compounds, the inhibitory rate was calculated by *I*% = (*A* − *B*)/*A* × 100%; where *A* was the peak area of HA without adding ACE inhibitors; *B* was the peak area of HA with adding ACE inhibitors.

### 2.6. Statistical Analysis

Unless otherwise indicated, all data were average of three repeats, and data are presented as means ± SEM. *Two-way* ANOVA and *t*-tests were employed in this paper, which were contained in SAS statistical analysis software, version 9.0 (SAS Institute, Cary, NC, USA).

## 3. Results and Discussion

### 3.1. Selection of Detectable Wavelength

**Figure 1:**
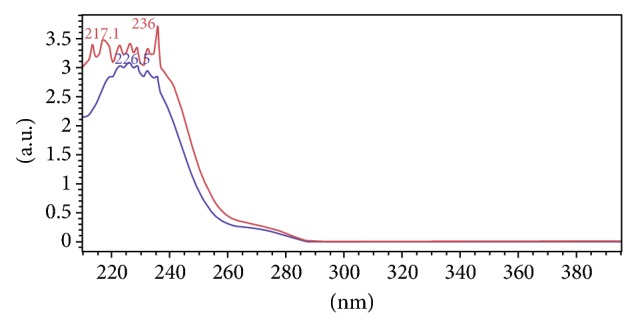
The ultraviolet absorbance spectra of HHL and HA.

5 mM HHL and 1 mM HA were detected at HPLC elution conditions as described in Section 2.3 from 210 nm–400 nm, respectively. At 226.5 nm, the absorbance of HA was the highest as shown in Figure 1 and HHL exhibited the wider absorbance from 217 nm to 236 nm. The absorbance ratio of HA was increased 1.62% at 226.5 nm than 228 nm, while the absorbance ratio of HHL was decreased 1.75% at 226.5 nm than 228 nm. As a result, we employed 226.5 nm as the detectable wavelength of the following HPLC analysis (see Figure 1).

### 3.2. Separations of the Targets

**Figure 2:**
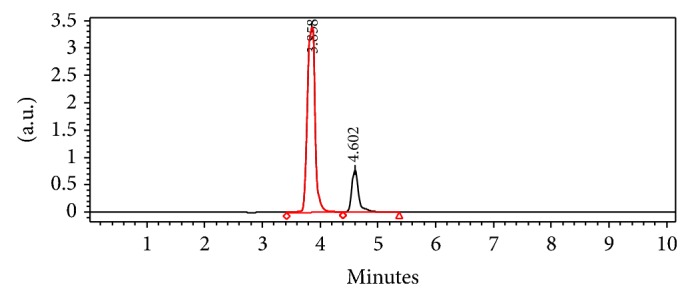
The separation result between HHL and HA by the RP-HPLC conditions.

Under the HPLC analytical conditions described in Section 2.3, the retention time of HHL and HA were 3.858 min and 4.602 min, respectively.  Figure 2 showed that HHL and HA were satisfactorily separated.

The separation rate was the separated level of the two borders upon peak; it was expressed as

(1) $$\begin{array}{c} R=2\frac{t_{\text{RB}}-t_{\text{RA}}}{W_{A}+W_{B}}. \end{array}$$

(Note: *R*: resolution; *t*_RB_: retention time of component HA; *t*_RB_: retention time of component HHL; *W*: base line width of peak.)

Figure 2 gave the result of “*R* = 1.48,” and *R* = 1 means the two peaks were separated elementary, and *R* = 1.5 means the two peaks were separated thoroughly. So, our results showed that the two targets had been separated satisfactorily (see Figure 2). 

### 3.3. The Linearity and the Lowest Detect Capability

1 mM, 0.5 mM, 0.1 mM, 0.05 mM, and 0.01 mM concentrations of HA were prepared as the high concentration of standard criterion for the linearity analysis; 5 *μ*M, 1 *μ*M, 0.5 *μ*M, 0.1 *μ*M, and 0.05 *μ*M concentrations of HA were used as the middle concentration of standard criterion for the linearity analysis; 0.1 *μ*M, 0.05 *μ*M, 0.01 *μ*M, 0.005 *μ*M and 0.001*μ*M were employed as lowest concentrations of standard criterion for the linearity analysis.

The results of the linearity analysis at the high concentration was *y* = 11947*x* + 1173.5, *R* ^2^ = 1; at middle concentration linearity: *y* = 11497*x* + 3886.3, *R* ^2^ = 1; at the lowest concentration: *y* = 45563*x* + 452.7,  *R* ^2^ = 0.939; at the concentration of 0.01 *μ*M, the linearity of peak area and concentration was accorded to the equation very well, while at the concentration of 0.005 *μ*M, the peak area began to deviate the equation. So, the lowest detect capability of this HPLC method was considered as 0.005 *μ*M.

 The method of Wu et al. [14] gave a linearity of HA from 0.01 mM to 1.0 mM (*y* = 1 × 10^7^ *x* + 1801.1, *R* ^2^ = 0.9998), but did not provide the lowest detect capability of the method. Also, the HA concentrations used in their method were in the high concentration linearity scope, but in fact, the linearity of middle and lowest concentrations was more valuable for ACE inhibitor assay.

### 3.4. Recovery Rate and Precision Effect

In order to detect the recovery rate, we used the method of sample recovery test. 5 mM, 2.5 mM, and 1 mM captopril were used as ACE inhibitors, and the HA concentration was detected in our HPLC conditions in the reaction system. 20 *μ*M HA was added to the reaction system, and the HA concentration was analyzed by HPLC again. The result of the recovery rate we got was 99.70%–100.46%, and the Relative Standard Deviation (RSD) was 0.38% (*n* = 9).

Otherwise, the HA concentrations of HHL and ACE reaction systems without ACE inhibitors were analyzed 9 times in three days, and the RSD result was 0.33%. The analysis of the HA peak area of these 9 times showed that there was no significant difference among them (*P* < 0.001). This proved that the method was precise and steady.

### 3.5. The Optimal Quantity Relationships between HHL and ACE

The selectivity and sensitivity were very important to the assaying method [22]. At the same time, other reaction conditions, such as Km, also affected the performance of the assay, because the method principle was based on the conversion rate of HHL to HA catalyzed by ACE. In this paper, the incubation time and temperature followed the values of Cushman and Cheung [15], and this method focused on the substrate transform rate from HHL to HA in different concentrations of HHL with a fixed concentration of ACE.

The total reaction volume was 100 *μ*L, including 10 *μ*L ACE in a concentration of 0.1 U/mL, different volume of HHL (1 *μ*L, 2 *μ*L, 4 *μ*L, 6 *μ*L, 8 *μ*L, 10 *μ*L, 20 *μ*L, 30 *μ*L, 40 *μ*L, 50 *μ*L) in a concentration of 5 mM, and the other volume of the system was filled up by 100 mM borate buffer (pH = 8.3). The reaction system was incubated at 37°C for 30 min, and 250 *μ*L HCl was added, and then the HPLC assay was performed.

**Figure 3:**
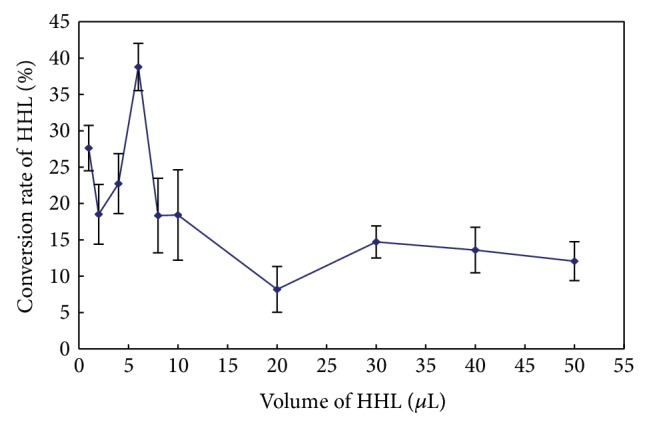
The effect of HHL concentration on the conversion rate from HHL to HA.

Figure 3 showed that, when the volume of HHL was 6 *μ*L, the HHL to HA was at the highest conversion rate 38.78%, and with the volume increasing of HHL, the conversion rate was decreased, which proved that accurate substrate quantity was helpful for improving the sensitivity of the HPLC method. When the HHL was 6 *μ*L, the HA concentration was 0.01119 mM, higher than 0.01 mM in the high concentration linearity which we got at the “linearity and lowest detect capability” section, and it was far higher than the lowest detection capability of the method we got. Thus, an optimal quantity relationship between HHL and ACE was 6 *μ*L HHL in concentration of 5 mM to 10 *μ*L ACE in a concentration of 0.1 U/mL; the amount in this method was far lower compared to the sample preparation conditions for HPLC analysis described by Wu et al. [14], who reported the total reaction volume was 70 *μ*L, made up of 50 *μ*L of 2.17 mM HHL, 10 *μ*L of 2 mU of ACE, and 10 *μ*L of different concentrations of ACE inhibitors. Because of the high sensitivity of this method, the usage of HHL could be economized. And it was rapid and convenient to facilitate the ACE inhibitory peptides research (see Figure 3).

### 3.6. The Verification of the Method with the Hydrolysates of Silkworm Pupae Protein

In order to verify the popularized potential in practice, we prepared silkworm pupae protein hydrolysates as ACE inhibitor and compared its ACE inhibitory activity with captopril (Table 1). The result showed that ACE inhibitory rate of hydrolyzates was 73.21 ± 2.17% in the concentration of 2 mg/mL, and inhibitory rate of captopril was 85.2 ± 1.13% in the concentration of 5 mM.

Comparison to the previous methods [20], the HPLC method developed in this paper separated HHL and HA perfectly in a more complex system and reduced analytical time (Figure 1). The advantages of this method are perhaps due to the different mobile components. In our study, the pH value of mobile phase was maintained at 2.9~3.3 with 0.05% TFA and 0.05% TTA in water. Different ratios of TFA and TTA will lead to the pH value deviate 2.9~3.3 the peak shape and separate rate of HHL and HA will be not ideal it showed the pH plays an important role in the system for RP-HPLC assay. The Iso-gradient elution time applied in our method was 10 min, which is longer than HA retention time 4.735 min the purpose was to elute the residues in the column more cleaner.

## 4. Conclusion

The HPLC assay described in this paper was a rapid, sensitive, and convenient method to determine the inhibitory activity on ACE. The results showed that HHL and HA could be separated completely with Iso-gradient elution program the assay effect and the lowest detection capability were improved, compared to the other methods. Also, this method skipped the extraction step of HA into ethyl acetate, which was indispensable in the method of Cushman and Cheung [15]. The results showed that this HPLC method could be used to explore new source of angiotensin I-converting enzyme inhibitory peptides rapidly and veraciously.

## Figures and Tables

**Table 1:** Recoveries of HA in reaction with different concentrations of captopril (*n* = 9).

Captopril	Original/(*μ*M)	Adding HA/(*μ*M)	Total (*μ*M)	Recovery rate (%)
5 mM	2.210 ± 0.012	20	22.143 ± 0.011	99.70
2.5 mM	3.713 ± 0.009	20	23.821 ± 0.014	100.46
1 mM	6.427 ± 0.016	20	26.441 ± 0.007	100.05

^*^All the assayed results were expressed as means ± SEM.

## Abbreviations

HHL: Hippuryl-_L_-histidyl-_L_-leucine;
HA: Hippuric acid;
ACE: Angiotensin I-converting enzyme;
TFA: Trifluoroacetic acid;
TTA: Triethylamine;
HPLC: High-performance liquid chromatography;
DAD: Photodiode array detector;
RAAS: Renin-angiotensin-aldosterone system.
